# The antiviral GTPase MxB is packaged into virions and binds via its N-terminal domain to alphaherpesvirus capsids

**DOI:** 10.1371/journal.ppat.1014370

**Published:** 2026-07-16

**Authors:** Sebastian Weigang, Manutea C. Serrero, Boris Bogdanow, Franziska Hüsers, Julia Lückel, Rudolf Bauerfeind, Fan Liu, Georg Kochs, Beate Sodeik

**Affiliations:** 1 Institute of Virology, Freiburg University Medical Centre, Faculty of Medicine, University of Freiburg, Freiburg, Germany; 2 Institute of Virology, Hannover Medical School, Hannover, Germany; 3 RESIST - Cluster of Excellence (EXC 2155), Hannover Medical School, Hannover, Germany; 4 Department of Structural Biology, Leibniz-Forschungsinstitut für Molekulare Pharmakologie, Berlin, Germany; 5 Institute of Virology, Charité - Universitätsmedizin, Freie Universität Berlin and Humboldt-Universität zu Berlin, Berlin, Germany; 6 Research Core Unit Laser Microscopy, Hannover Medical School, Hannover, Germany; 7 German Centre for Infection Research (DZIF), Hannover-Braunschweig Partner Site, Hannover, Braunschweig, Germany; Washington State University, UNITED STATES OF AMERICA

## Abstract

The interferon-inducible myxovirus resistance proteins (Mx) belong to the dynamin-like GTPases; MxA is a restriction factor against several RNA viruses, while MxB restricts infections of lentiviruses and herpesviruses. Humans express MxA(1–662) with an N-terminal domain (NTD) of 43 residues, MxB(1–715) with an NTD of 91 residues, and a truncated MxB(26–715) with an NTD of 66 residues. Although the roles of the GTPase and stalk domains during infection are increasingly being elucidated, the functions of the different NTDs remain poorly understood. Using cell lines stably expressing Mx proteins, we show that MxB(1–715), but not MxA, inhibited infection by herpes simplex virus (HSV-1) and pseudorabies virus (PrV). Quantitative mass spectrometry and subviral fractionation experiments indicate that MxB(1–715), but not the truncated MxB(26–715) or MxA(1–662), was enriched in the tegument of HSV-1 and PrV virions. Moreover, dimeric, recombinant, chimeric proteins with the NTD peptides MxB(1–91) or MxB(1–35), and to a lesser extent MxB(26–91), bound to tegumented HSV-1 capsids, while MxA(1–43) did not. In contrast, de-tegumented HSV-1 and PrV capsids bound to proteins with MxB(1–91), but not to those with MxB(1–35) or MxA(1–43). Our findings indicate that the NTD of MxB is crucial to restrict HSV-1 and PrV infections, that newly assembled virions package MxB into the tegument around the capsid, and that both the N-terminal MxB amino acid residues 1–25 and 26–91 contribute to its binding to tegumented and de-tegumented herpesviral capsids.

## Author summary

Infected cells release interferons that induce naïve cells to express hundreds of antiviral interferon-stimulated genes that, in concert, limit viral spread to the neighboring cells. MxB is one of many ISG-encoded proteins that restricts herpesviruses, likely by interfering with their capsid functions. In this study, we investigated how human MxB, but not the related MxA protein, inhibits infection by herpes simplex virus or pseudorabies virus. Furthermore, we unexpectedly discovered that progeny HSV-1 and PrV virions incorporated the full-length MxB(1–715) but not the N-terminally truncated MxB(26–715) or MxA proteins into the tegument layer surrounding the capsid. Biochemical assays show that distinct regions in the N-terminal domain of MxB (1–91) contribute to its binding to tegumented and de-tegumented herpesvirus capsids and to restricting alphaherpesvirus infections. Our study provides new insights into understanding how MxB can restrict the broad family of herpesviruses.

## Introduction

The myxovirus resistance proteins MxA and MxB, whose expression is upregulated by type I and type III interferons (IFNs), are antiviral members of the dynamin superfamily of GTPases that have a low nucleotide affinity but a high intrinsic GTPase activity [1–4]. Most mammals have two paralogous genes with about 60% sequence similarity: *MX1* encodes MxA and *MX2* encodes MxB [1,5,6]. Humans express MxB(1–715) of 78 kDa and a shorter isoform MxB(26–715) of 76 kDa, which are likely translated from the same mRNA, and one isoform MxA(1–662) of 76 kDa [7]. Cryoelectron tomography and crystal structures show that the Mx proteins share an extended architecture with a central bundle signaling element connecting the N-terminal GTPase domain to the C-terminal stalk, which in turn mediates the formation of dimers, tetramers, and ring-shaped, helical oligomers [8–11].

**Fig 1:**
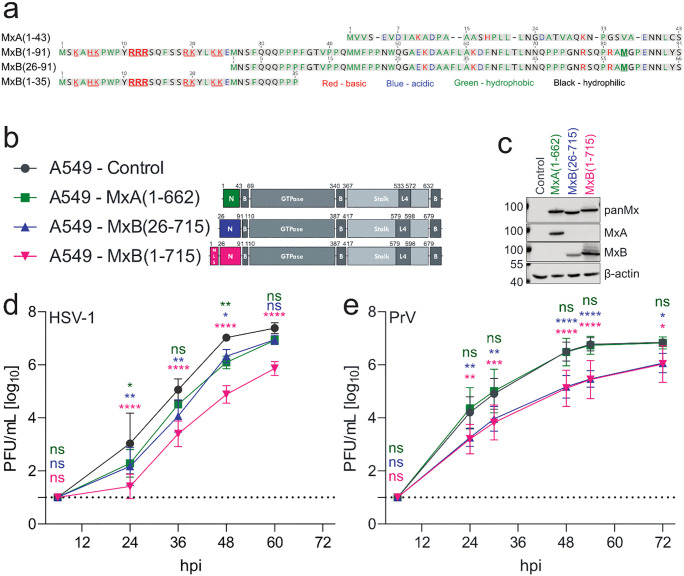
HSV-1 and PrV multistep-growth curves. **(a)** Amino acid residue alignment of human MxA(1-43) (Genbank accession number P20591), MxB(1-91) and MxB(26-91) (P20592) NTD sequences using the Geneious Alignment with the Blosum62 matrix. Residues were color-coded according to their biochemical properties. **(b)** Domain organization of the MxA-NTD (green), MxB(26-715)-NTD (blue), and MxB(1-715)-NTD (red), as well as the common bundling signaling elements (B), the GTPase domains, and the stalks with the L4 loop domains. **(c)** Immunoblot of A549 cells expressing different Mx proteins or control cells using panMx-specific antibodies (M143) and MxA-specific or MxB-specific polyclonal antisera. **(d and e)** A549 cells expressing MxB(1-715) (red), MxB(26-715) (blue), MxA(-662) (green), or un-transduced cells (black) were infected with HSV-1 at an MOI of 0.001 (d) or with PrV at an MOI of 0.0001 **(e)**. The mean titers of the culture supernatants were determined by plaque assay. The error bars represent the SD from independent biological replicates (4 for HSV-1; 6 for PrV). The dotted lines indicate the detection limit. Two-way ANOVA tests with Dunnett multiple comparison comparing the log-transformed titers of cells expressing Mx proteins to the un-transduced A549 control cells. *, p < 0.05; **, p < 0.01; ***, p < 0.001; ****, p < 0.0001; ns, nonsignificant.

Mx proteins have unique flexible regions, namely the N-terminal domains (NTDs; c.f. Fig 1a) of MxA(1–43), MxB(1–91) and MxB(26–91), and the L4 loops of MxA(533–572) and MxB(579–598) in the stalk domains (Fig 1b). The NTDs lack any predicted structural elements, but the 25-residue-long N-terminal extension (NTE) of MxB(1–715) contains a bipartite, nuclear localization sequence (NLS) that contributes to its nuclear pore complex (NPC) binding [7,12–14]. Upon basal expression, MxB(1–715) is localized at the cytoplasmic face of the NPCs, but upon IFN induction, it is also increasingly found in the nucleoplasm and cytoplasmic biomolecular condensates [7,12,15–18]. Recent yeast-2-hybrid, co-immunoprecipitation and protein cross-linking data show that MxB(1–715) can bind to several nucleoporins (NUPs), including NUP358 (RanBP2), NUP214, NUP98, NUP88, NUPL2, and RAE1 which face the cytosol [14,18,19].

While the better-characterized human MxA inhibits a broad spectrum of RNA viruses, like myxoviruses and bunyaviruses, as well as hepatitis B virus, it was reported only about 15 years ago that human MxB also possesses antiviral activity and perturbs the replication of human immunodeficiency virus 1 (HIV-1) and other primate lentiviruses [20,21]. Later, we and others reported that MxB also restricts the alphaherpesviruses herpes simplex virus 1 (HSV-1), HSV-2, the betaherpesviruses human and murine cytomegaloviruses (HCMV, MCMV), and the gammaherpesviruses Kaposi sarcoma-associated herpesvirus (KSHV) and murine herpesvirus MHV-68 [16,17,22–25]. Moreover, MxB restricts the hepatitis C virus and hepatitis B virus [26–28].

Lentiviruses and herpesviruses enter cells by fusion of their envelopes with host membranes. Their incoming cytosolic capsids travel along microtubules to the nucleus and dock on the cytoplasmic filaments of the NPCs for genome release into the nucleoplasm and viral transcription and replication (reviewed in [29–32]. Human MxB impedes incoming HIV-1 capsids before docking on the NPCs and genome integration into the host chromosomes, but after viral fusion and reverse transcription [14,15,20,21,33,34]. Similarly, human MxB perturbs incoming HSV-1 capsids after viral fusion but before docking on the NPCs and genome release into the nucleoplasm [16,18,24]. The cytosolic, MxB-containing biomolecular condensates trap incoming HIV-1 and HSV-1 capsids [18]. Thus, MxB interferes with intracellular lentivirus and herpesvirus capsid transport along microtubules, capsid interactions with the NPCs, and the import of incoming viral genomes from the capsids through the NPCs into the nucleoplasm.

HIV-1 restriction requires MxB dimerization and oligomerization, and MxB loses its antiviral activity when the 25 N-terminal residues are missing or when the triple-Arg motif (11RRR13; Fig 1a) in the NLS is mutated to Ala [9–11,21,35,36]. Moreover, a chimeric protein of the MxB-NTD(1–91) fused to MxA(43–662) restricts HIV-1 with a similar effectivity as MxB(1–715) [37]. MxB binds to HIV-1 capsids through its NTD, but HIV-1 strains with mutations in the capsid protein evade the MxB restriction without interfering with its binding to capsids [10,15,34,38]. In cells, the MxB condensates trap incoming HIV capsids, thereby preventing their targeting to the NPCs and entry of viral genomes into the nucleoplasm [18].

As for HSV-1, MxB dimerization, oligomerization, and the first 25 residues of its NTD are required for efficient restriction at a low multiplicity of infection (MOI) [16,18,24]. Moreover, a chimeric protein comprising MxB-NTD(1–85) fused to MxA(38–662) perturbs HSV-1 infection, although not as efficiently as the authentic MxB(1–715) [24]. MxB residue M83, encoded by the predominant human MX2 allele (Fig 1a) but absent in minor human variants or other primates, is critical for restricting HSV-1 infection [17]. In contrast to the restriction mechanisms against HIV-1, GTP binding and hydrolysis also contribute strongly to MxB’s activity against alphaherpesviruses [16,18,24].

We have reconstituted functional capsid-host protein complexes in cell-free assays [39–42]. Using cytosolic extracts containing different Mx proteins, we recently showed that MxB(1–715) but not MxA(1–662) co-sediments with tegumented and de-tegumented HSV-1 capsids; however, increasing amounts of associated tegument proteins reduce the binding of MxB(1–715) to the capsids [42]. Moreover, immunoelectron microscopy has demonstrated direct binding of MxB(1–715), but not of MxA(1–662), to the capsids, while the truncated MxB(26–715) binds to a lesser extent [42]. In cytosolic extracts containing ATP/GTP, both MxB(1–715) and MxB(26–715) induce capsid deformation, complete capsid disassembly, and the release of the viral genomes, while MxA(1–662) does not do this [42]. Using HSV-1 inocula with CLICKable genomes for fluorescence microscopy, we recently showed that MxB(1–715) also induces premature genome release from incoming HSV-1 capsids in MxB-expressing cells [18]. While these findings establish MxB as a potent effector that recognizes HSV-1 capsids and triggers their disassembly, the molecular determinants of this interaction remain unclear. It is not known which MxB regions contribute to capsid recognition, whether the NTD alone suffices, or whether MxB capsid binding could lead to its incorporation into virions. Addressing these questions is essential to understanding how MxB can restrict the broad family of herpesviruses.

Here, we show that MxB(1–715) also limited infection of pseudorabies virus (PrV), a swine alphaherpesvirus, but, in contrast to HSV-1, the truncated MxB(26–715) also restricted PrV infection as effectively as the full-length MxB(1–715). Late in infection, MxB(1–715) but not MxB(26–715) colocalized with HSV-1 progeny capsids in the cytosol. We have analyzed HSV-1 particles released from Mx protein-expressing cells by mass spectrometry, immunoblotting, and immunoelectron microscopy and show that MxB(1–715), but neither MxB(26–715) nor MxA(1–662), was enriched in the tegument of HSV-1 and PrV virions. Recombinant chimeric proteins with the MxB(1–91) NTD bound to tegumented and de-tegumented HSV-1 capsids. Future studies need to address whether the incorporated MxB reduces the specific infectivity of the virions released from interferon-induced, infected cells, and thereby potentiates its antiviral function.

## Results

### MxB protein restricts infection of the alphaherpesviruses HSV-1 and PrV

To further characterize the impact of Mx proteins on herpesviral infections, we used human lung epithelial A549 cells expressing MxB(1–715), MxB(26–715), or MxA(1–662) under the control of a constitutively active HCMV promoter [24] (Fig 1b). Immunoblots using an antibody directed against a conserved epitope in the GTPase domain showed that the cell lines expressed comparable levels of the respective Mx proteins (Fig 1c, anti-panMx).

A549-MxB(1–715) cells restricted the release of infectious BAC derived HSV-1 strain 17^+^ virions by about 100-fold in a multi-step-growth curve when compared to the mock-transduced cells (Fig 1d). Interestingly in contrast to HSV-1 strain McIntyre [24], the release of infectious virions of the BAC derived HSV1(17^+^)Lox was also delayed in cells expressing the truncated MxB(26–715) or MxA(1–662) when compared to the mock-transduced cells (Fig 1d). MxB(1–715), and interestingly also the truncated MxB(26–715), but not MxA restricted the release of infectious pseudorabies virus (PrV), a porcine alphaherpesvirus, by about 100-fold (Fig 1e).

**Fig 2:**
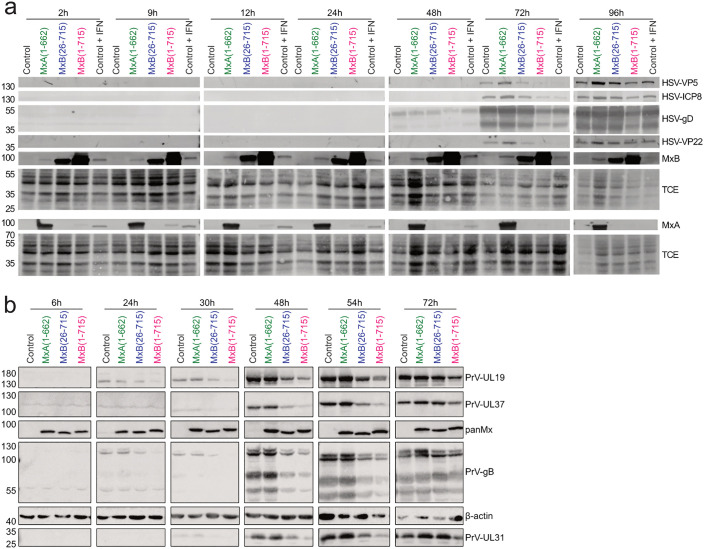
HSV-1 and PrV protein expression. **(a)** A549 cells were infected with HSV-1 at an MOI of 0.001 and harvested at the indicated hpi. One of the two control cell wells had been pre-treated with 1000 U/ml IFN-α a2 for 24 **h.** The cell lysates were analyzed by immunoblot using antibodies against MxA, MxB, the capsid protein VP5 (pUL19), the ssDNA-binding protein ICP8, the glycoprotein gD, or the tegument protein VP22. As a loading control, the proteins on the membrane were detected by UV activation of 2,2,2-trichloroethanol (TCE). Molecular weight markers are indicated in kDa on the left. **(b)** A549 cells were infected with PrV at an MOI of 0.0001 and harvested at the indicated hpi. The cell lysates were analyzed by immunoblot using antibodies against Mx proteins (M143), the capsid protein VP5 (pUL19), the glycoprotein gB, the tegument protein pUL37, or the nuclear egress complex protein pUL31. The host protein actin was used as a loading control. Molecular weight markers are indicated in kDa on the left.

Accordingly, the expression of capsid protein HSV1-VP5, ssDNA binding protein HSV1-ICP8, envelope protein HSV1-gD, and tegument protein HSV1-VP22 were reduced in cells expressing MxB(1–715) but not in A549-MxB(26–715), A549-MxA, or A549 control cells (Fig 2a). In contrast, the expression of capsid protein PrV-VP5 (pUL19), tegument protein PrV-pUL37, envelope protein PrV-gB, and nuclear egress protein PrV-pUL31 was reduced in cells expressing either MxB(1–715) or MxB(26–715) when compared to A549-MxA or A549 control cells (Fig 2b). Throughout HSV-1 (Fig 2a) or PrV (Fig 2b) infection, the transduced cells stably expressed MxA(1–662), MxB(26–715), or MxB(1–715). An IFN-α pre-treatment for 24 h of the A549 control cells also induced expression of MxA and MxB (1–715), which remained elevated up to 48 hpi but was reduced at 72 hpi with HSV-1 at a low MOI (Fig 2a). Although IFN-α led to a lower induction of Mx protein expression, together with the hundreds of other interferon-stimulated genes [43–45], the HSV-1 infection was inhibited as well as by a high expression of MxB(1–715) alone.

**Fig 3:**
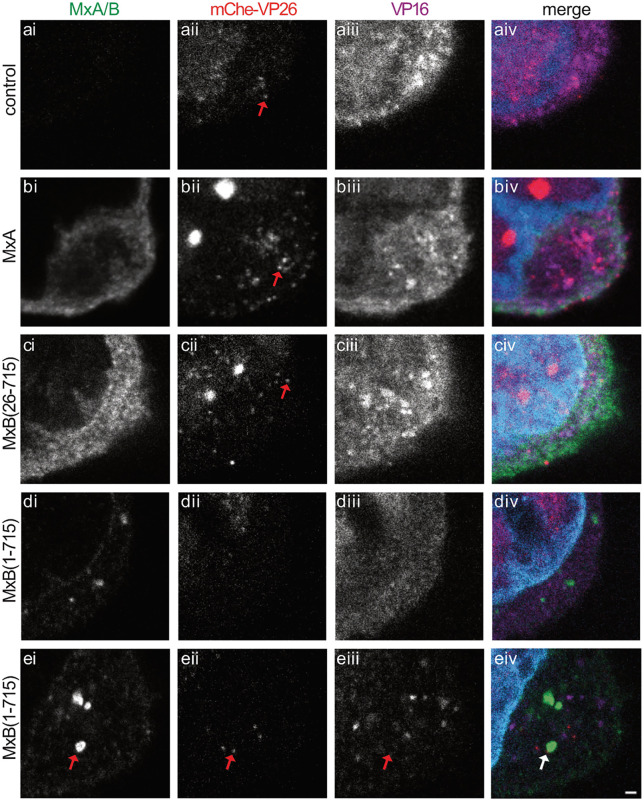
Subcellular localization of Mx proteins during HSV infection. A549 cells were infected with HSV1(17^+^)Lox-CheVP26 at an MOI of 20, fixed at 9 hpi, and labeled for MxA or MxB (green), tegument protein VP22 (purple), and DNA (blue). Representative images of cells expressing empty control, MxA, MxB(1-715), or MxB(26-715). The arrows indicate single progeny capsids in the cytoplasm and a close association between MxB and progeny, cytoplasmic capsids. Panels **(d)** and **(e)** show two representative examples of cells expressing MxB(1‑715) illustrating the heterogeneous infection kinetics observed under this condition. Scale bar: 1 µm.

Next, we determined the subcellular localization of the Mx proteins during HSV-1 infection. The A549 cells were infected with HSV1-CheVP26 at an MOI of 20 for 9 h. HSV1-CheVP26 has a Cherry tag on the small capsid protein VP26 to trace individual incoming and progeny capsids in the cytoplasm and the nucleus [46–49]. MxA(1–662) and MxB(26–715) were diffusively localized throughout the entire cytoplasm (Fig 3bi and 3 ci). In contrast, MxB(1–715) was enriched in cytoplasmic biomolecular condensates and at the nuclear envelopes (Fig 3di and 3ei). Similar MxB(1–715) biomolecular condensates trap incoming HSV-1 capsids early in infection [18]. While there were many cells with nuclear and cytoplasmic progeny capsids in the control, MxA, and MxB(26–715) A549 cells, there were fewer infected MxB(1–715) A549 cells, and those contained fewer cytoplasmic capsids (Fig 3). Accordingly, the expression of HSV1-VP16 (Fig 3iii) was lower in the MxB(1–715) than in the other A549 cell lines. Also HSV1-gD expression was reduced in cells expressing MxB(1–715) (S1fiii Fig), but to a lesser extent than VP16. The progeny HSV-1 capsids did not co-localize with MxA or the truncated MxB(26–715), but in a fraction of cells, there were cytoplasmic capsids in close proximity to the cytoplasmic MxB(1–715) biomolecular condensates.

Next, we monitored the induction of interferon-stimulated genes (ISGs) in A549 control cells following IFN-α pretreatment or HSV-1 infection at a low MOI. At 24 hpi and even more so at 48 hpi, the transcript for HSV1-ICP0 (infected cell protein 0) was strongly expressed but not detected in uninfected A549 cells (S2a Fig). The IFN-α treatment but not HSV-1 infection induced the expression of *IRF-7* (S2b Fig), *ISG-15* (S2c Fig), *MX1* (S2d Fig), and *MX2* (S2e Fig). Accordingly, IFN-α treatment but not HSV-1 infection also led to MxB and MxA protein expression (S2f Fig). In contrast, both IFN-α treatment and HSV-1 infection induced moderate IFN-β expression (S2g Fig), while the IL-6 levels remained constant under all conditions (S2h Fig).

In summary, HSV-1 infection at low MOI did not induce ISG expression *per se* and, importantly, it also did not induce the expression of endogenous MxA or MxB. However, A549 cells expressing recombinant MxB(1–715) restricted HSV-1 and PrV infection at low MOI, as shown in multiple-step growth curves. Therefore, these A549 cell lines are well-suited for characterizing distinct Mx effector functions on herpesviral infections.

### MxB but not MxA is packaged into HSV-1 particles

Herpesvirus-infected cells release a wide variety of viral structures. The most prominent are heavy H-particles, which include complete virions with genomes, capsids, tegument, and envelopes, and light L-particles, which contain viral envelopes and tegument but neither capsids nor viral genomes [50–55]. As MxB(1–715) restricts HSV-1 infection in multi-step infection rounds [16,18,24] (Fig 1d), we investigated whether the elevated MxB expression changed the protein composition of viral particles released from these cells. We harvested particles at the late stage of infection secreted from A549-MxA(1–662) or A549-MxB(1–715) cells infected with HSV-1 at a low MOI and used sedimentation velocity centrifugation on glycerol-tartrate density gradients to separate the L and H particle fractions. Using label-free quantitative shotgun mass spectrometry, as reported previously for HCMV [54], we compared the proteomes of viral particles harvested from MxA- or MxB-expressing cells with the total proteome of the respective cell line.

**Fig 4:**
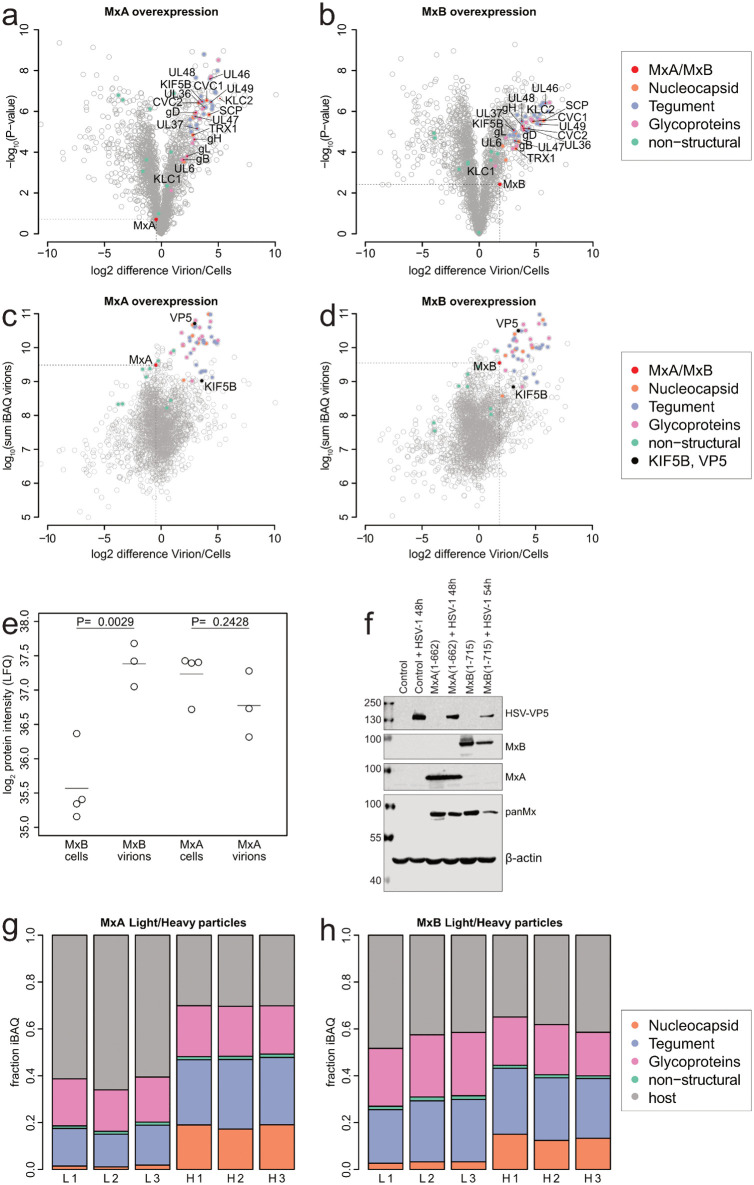
Mass spectrometric protein analysis of extracellular HSV-1 particles. Protein composition of L-particle and H particles harvested from infected A-549-MxA(1-662) or A549-MxB(1-715) cells. Cells were infected with an MOI of 0.001 with HSV-1 for 56h. **(a, b)** Relative protein quantification of H-particles secreted from **(a)** MxA(1-662)-A5439 or **(b)** A549-MxB(1-715) expressing cells compared to the respective whole cell lysates. In addition to the highlighted Mx proteins (red), several categories of viral and host proteins were color-coded. Means of fold-change differences and P values of two-sided t-tests without multiple hypothesis correction were based on quadruplicate measurements for cell lysates and triplicate measurements for H particles. Quantification was performed using the LFQ algorithm in MaxQuant, and only unique peptides were used. **(c, d)** Absolute quantification of the proteins of H-particles secreted from **(c)** MxA(1-662)-A5439 or **(d)** A549-MxB(1-715) expressing cells, compared to the respective total cell lysates based on the intensity-based absolute quantification (iBAQ)-values that were summed up across three replicates, compared to enrichment levels. **(e)** Log2-transformed protein intensity (LFQ) of the proteins of values in the respective MxA(1-662) or MxB(1-715) overexpressing and HSV-1 infected cells or H particles secreted from MxA(1-662)-A5439 or A549-MxB(1-715) expressing cells compared to the respective whole cell lysates, with the individual dots indicating all replicates and the lines indicating the respective mean values. The p-values are based on two-sided t-tests. **(f)** Control, MxA, or MxB overexpressing A549 cells were left untreated or infected with HSV-1 at an MOI of 0.001 for 48 or 54h. Immunoblot of total cell lysates probed with antibodies directed against the Mx GTPase domain (panMx M143), MxA, MxB, or VP5 (pUL19). Actin was used as a loading control. Molecular weight markers are indicated on the left in kDa. **(g, h)** The iBAQ-values for the protein compositions of L- and H-particles secreted from MxA(1-662) **(g)** or MxB(1-715) **(h)** expressing A549 cells infected with HSV-1 were analyzed for all biological replicates. The color code indicates different categories of viral and host proteins.

Regardless of whether A549-MxA (Fig 4a) or A549-MxB cells had assembled the particles (Fig 4b), the H fractions were enriched for HSV-1 structural proteins over the respective cell lysates. Examples are major capsid proteins VP5 (pUL19) and VP19c (pUL38; TRX1), minor capsid proteins pUL17 (CVC1), pUL25 (CVC2), and pUL6, minor tegument proteins pUL36 and pUL37, major tegument proteins VP11/12 (pUL46), VP16 (pUL48) and VP22 (pUL49), and envelope proteins gB, gD and gH (Fig 4a, 4b). In contrast, non-structural HSV-1 proteins, e.g., the subunits of DNA helicase-primase pUL5, pUL8, and pUL52, or the DNA polymerase pUL30 and pUL42, were not enriched. In addition, herpesviruses package a variety of host proteins [54,56–58]. As reported [54,56–58], we also detected the heavy chain subunit KIF5B of the microtubule motor kinesin-1, and in addition, also its light chain subunit KLC2 in the particles secreted from either cell line. Intriguingly, the H particles secreted from the respective cells were also enriched for MxB (Fig 4b) but not for MxA (Fig 4a).

Next, we calculated the intensity-based absolute quantification (iBAQ) values to estimate the absolute levels of individual proteins in these samples [42,54,59]. As expected, the HSV-1 structural capsid (orange in Fig 4), tegument (blue in Fig 4), and envelope (pink in Fig 4) proteins were the most abundant proteins in H-particles secreted from either A549-MxA (Fig 4c) or A549-MxB (Fig 4d) infected cells. While both MxA from A549-MxA cells (red Fig 4c) and MxB from A549-MxB cells (red in Fig 4d) could be detected, MxB was enriched by about 4-fold in H-particles over the respective cell lysates, while MxA was not. The log-transformed protein intensity (LFQ) data of the biological replicates show that HSV-1 virions were significantly enriched for MxB but not for MxA when compared to the corresponding A549 cell lysates (Fig 4e). As for the PrV infection (c.f. Fig 2b), immunoblot analyses of the A549 lysates confirmed that the HSV-1 infection had not increased the expression of MxA or MxB (Fig 4f). Intriguingly, the L-particles from the A549-MxA cells contained a larger portion of host proteins than the corresponding H-particles (grey in Fig 4g). In contrast, the magnitude of host proteins was similar in L- and H-particles from the A549-MxB cells (grey Fig 4h). As reported before, the L particles contained only low amounts of HSV-1 capsid or non-structural proteins, irrespective of whether the cells expressed MxA (Fig 4g) or MxB (Fig 4h).

These data demonstrate the successful separation and harvesting of HSV-1 H and L particles by the velocity gradient centrifugation and show that these fractions contained few detached cells. Moreover, they show that MxB was enriched in the H fraction, arguing for a specific incorporation of MxB but not of MxA into virions.

### HSV-1 and PrV virions package MxB(1–715) into the tegument

**Fig 5:**
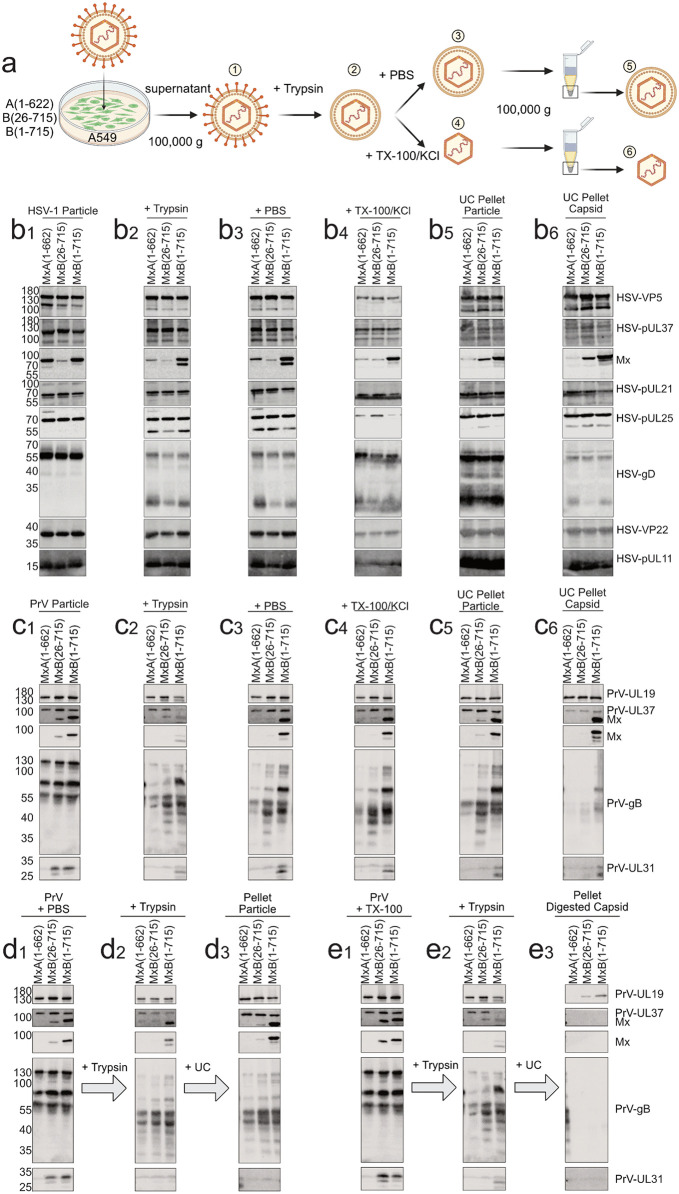
MxB(1-715) is packaged into the tegument of HSV-1 and PrV virions. **(a)** Workflow of the particle fractionation and capsid preparation: Extracellular particles were harvested from A549 cells expressing MxA(1-662), MxB(26-715), or MxB(1-715) and infected with HSV-1 or PrV resuspended in an equal volume of PBS (1), and trypsin was added to digest any proteins attached to the particle surfaces, followed by the addition of trypsin inhibitor (2). The particles were kept in PBS (3) or lysed with 1% TX-100 and 0.5 M KCl (4). The lysates were layered on top of a 30% sucrose cushion in PBS and centrifuged to sediment the capsids and capsid-associated proteins (5) or treated with 1% TX-100 at 0.5 M KCl (6). Samples from each experiment and each preparation step were resuspended in SDS sample buffer. Created with BioRender.com and licensed under CC BY 4.0 (https://BioRender.com/wwaswae). **(b and c)** HSV-1 particle fractions containing 2 × 10^8^ pfu (b) or PrV particles containing 4 × 10^7^ pfu (c) were used as starting material (1). Lysates of the initial purified, extracellular particles (1), the trypsin-treated particles (2), the PBS-treated particles before (3) and after (5) ultracentrifugation at 100,000 x g, and the Triton/KCl-treated particles before (4) and after (6) ultracentrifugation were separated by SDS-PAGE and probed with antibodies directed against the Mx GTPase domain (panMx M143), the major capsid protein VP5 (pUL19), the glycoproteins gD and gB, as well as the tegument proteins pUL36, pUL37, VP22, pUL11 and pUL31. **(d and e)** MxB(1-715) is protected from trypsin digestion in viral particles. Purified extracellular particles of PrV (4 x 10^7^ pfu, as described in panel c) were (d1) mock-treated (d1) or treated with 1% Triton X-100 in PBS (e1). Then the mock-treated (d2) and Triton-treated (e2) particles were incubated with trypsin for 30 min at 37°C. The digestion was stopped by the addition of a trypsin inhibitor. Finally, the mock- or trypsin-treated particle fractions were ultracentrifuged, and the resulting pellets of the mock/trypsin (d3) or Triton/trypsin (e3)-treatments were resuspended in SDS sample buffer, the lysates were separated by SDS-PAGE, and probed with antibodies as described for panel **c.** The panels show representative results of (b) three and (c, d, e) two independent experiments.

Next, we fractionated extracellular viral particles to determine the subviral localization of MxB. The particles secreted from A549 cells expressing MxA(1–662), MxB(26–715), or MxB(1–715) and infected with HSV-1 or PrV for about 3 days were harvested by ultracentrifugation (Fig 5a, step 1). The particles were treated with trypsin to dissociate any host or viral proteins bound to their outer surface that were not protected by the virion envelopes or other vesicular membranes. To stop the proteolysis, protease inhibitors were added after 30 min at 37°C (step 2). One-half of each sample was combined with an equal volume of PBS to preserve host and virion membrane integrity (step 3). The other half was mixed with an equal volume of two-fold lysis buffer containing 2% TX-100 and 1 M KCl (step 4). The samples sedimented from the PBS treatment by ultracentrifugation (step 5) included all particles as before (step 2, step 3). In contrast, the detergent/salt treatment with TX-100 and KCl solubilizes viral envelopes, vesicle membranes, and their proteins and weakens intra-tegument protein-protein interactions, leading to some dissociation of outer tegument proteins from the capsids [39,41,42,60]. While envelope and outer tegument proteins were released, inner tegument proteins remained capsid-associated and were sedimented into the pellet fractions upon ultracentrifugation (step 6). All samples of the particle fractionation procedure were analyzed by immunoblot.

Similar amounts of the HSV-1 proteins VP5, pUL25, pUL11, pUL21, and pUL37 (Fig 5b), or the PrV proteins pUL19, pUL37, and gB (Fig 5c) were present in the respective samples from the cells expressing MxA(1–662), MxB(26–715), or MxB(1–715), allowing a comparison among the cell lines based on their capsid amounts. However, based on VP5, the yield of capsids varied among the different cell lines and also the particles from different fractionation steps from one cell line contained varying amounts of the capsid proteins HSV1-VP5 (e.g., b1 versus b4) or PrV-pUL19 (e.g., c1 versus c2), and therefore different capsid yields as baselines.

The trypsin treatment generated a truncated form of the HSV-1 minor capsid protein pUL25 (b2, b3), indicating that the extracellular particles comprised a significant fraction of capsids that were not protected by envelopes, as reported previously by Döhner et al., 2006 [52]. However, the truncated pUL25 was released from the capsids, as it did not co-sediment with the capsids during ultracentrifugation (b5). The outer tegument protein VP22 was also somewhat susceptible to trypsin digestion (b1, b2), and the TX-100/KCl solubilization reduced it further (b4, b6). In contrast, the envelope protein gD, with a large domain extruding from host and viral membranes, was susceptible to trypsin digestion and was reduced compared to the starting material (b1).

Similarly, trypsin had digested most of the particle-associated MxA(1–662), indicating that it had been associated with particle surfaces rather than packaged into virions (b2 versus b1). Some, but not all, particle-associated MxB(1–715) was susceptible to trypsin digestion, indicating that some of it had also been stuck on the particle surfaces (b2 and b3 versus b1). However, this smaller fragment derived from MxB(1–715) did not co-sediment with the particles or capsids (b5 and b6). In contrast, the full-length MxB(1–715) remained associated with the capsids, like the tegument proteins pUL37, pUL21, VP22, and pUL11 (step 5 and 6). Compared to MxB(1–715), the extracellular particles contained less MxB(26–715), but MxB(26–715) remained capsid-associated after the TX-100/KCl treatment (Fig 5b6).

Similarly, the PrV samples from cells expressing MxA(1–662), MxB(26–715), or MxB(1–715) contained throughout the six treatment steps similar amounts of the major capsid protein pUL19 (VP5 in HSV-1) and the tegument protein pUL37 (Fig 5c1-c6). The envelope protein gB was susceptible to trypsin digestion (c1, c2). In contrast to HSV1-gD, PrV-gB has a long cytoplasmic domain that is protected within virions and vesicles (c2, c3, c4, and c5) but solubilized from the capsids upon TX-100/KCl treatment (c6). In particles from MxB(1–715) or MxB(26–715) but not from MxA expressing cells, the peripheral subunit pUL31 of the nuclear egress complex was detected (c1). pUL31 was susceptible to trypsin digestion (c2) and mostly absent from the final capsid preparation (c6). The presence of pUL31 suggests that the extracellular PrV particle preparations from A549-MxB cells contained host membranes and debris to some extent.

Notably, we needed more infected A549-MxB cells than A549-MxA cells to harvest similar amounts of extracellular particles, likely because MxB restricts infection. Like for the HSV-1 particles, we detected high levels of MxB(1–715), but unlike HSV-1, only little MxB(26–715) and no MxA(1–662) in the extracellular PrV particle fractions (c1). As for HSV-1, the full-length MxB(1–715), but unlike HSV-1, not the truncated MxB(26–715), remained associated with the PrV capsids throughout the fractionation (Fig 5c6).

To test whether capsid-associated MxB is in principle susceptible to trypsin digestion, we treated PrV particles resuspended in PBS (Fig 5d1) or TX-100/KCl (e1) with trypsin (d2, e2). After ultracentrifugation, the particles in PBS (d3) still contained MxB(1–715), while the sedimented capsids treated with trypsin after the Tx-100/KCl solubilization did not contain MxB (e3). These experiments demonstrate that MxB(1–715), but not MxA, had been packaged into the tegument of HSV-1 and PrV virions during capsid envelopment in the cytoplasm.

### MxB but not MxA epitopes become accessible in HSV-1 virions after envelope rupture by osmotic shock

**Fig 6:**
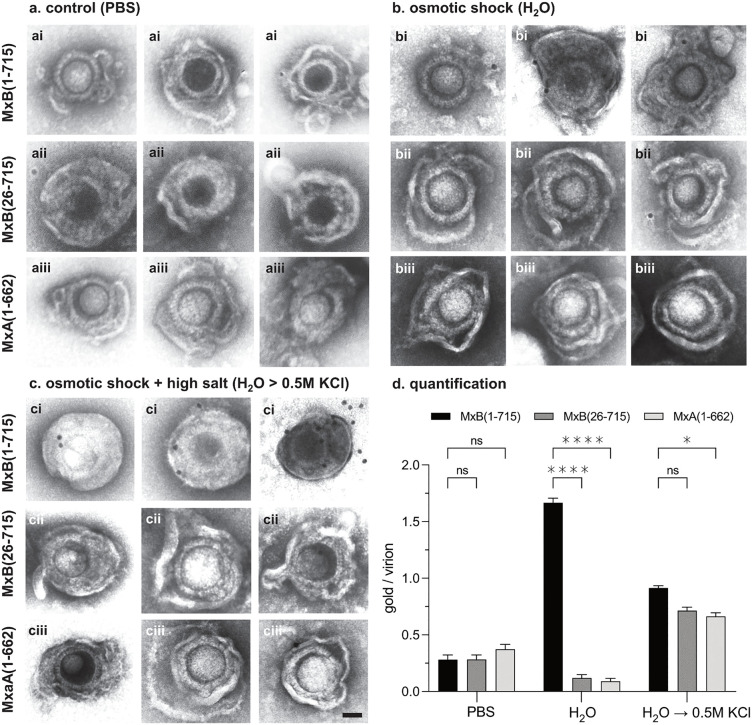
Detection of MxB in HSV-1 particles by immuno-EM. **(a to c)** Extracellular particles were harvested from A549 cells expressing MxA(1-662), MxB(26-715), or MxB(1-715) and infected with HSV-1, resuspended in PBS, treated with trypsin, and absorbed on EM grids. The samples were left untreated in PBS (a; PBS- > PBS), or the viral envelope was opened by osmotic lysis in pure H_2_O. Then the lysed particles were incubated in PBS (b; H_2_O > PBS) or with 0.5 M KCl (c; H_2_O > high salt) for 30 min at RT. All specimens were processed for immunogold labeling analysis using the pan-Mx-specific M143 antibody. Scale bars represent 50 nm. **(d)** The mean number of protein A-gold particles within a 20 nm distance of 100 randomly documented virions per condition, collected across 3 technical replicates, was determined. The error bars represent the SEM. Kruskal-Wallis test with Dunn’s multiple comparison: *, p < 0.05; **, p < 0.01; ***, p < 0.001; ****, p < 0.0001; ns, not significant.

Next, we investigated the incorporation of Mx proteins into virions by quantitative immunoelectron microscopy. Extracellular HSV-1 particles harvested from the Mx-expressing cells were treated with trypsin to remove any Mx proteins associated to the particle surfaces and adsorbed onto EM grids. They were then incubated in PBS (PBS → PBS → PBS; Fig 6a), in H_2_O to induce an osmotic shock (PBS → H_2_O → PBS, Fig 6b), or in H_2_O and then high salt buffer (PBS → H_2_O → 0.5 M KCl → PBS, Fig 6c). All specimens on the EM grids were incubated with the generic anti-Mx antibodies (M143) diluted in PBS, followed by colloidal gold particles coated with protein A that binds antibodies at their Fc domains and negative staining as reported before [40,42]. We reasoned that in PBS, the viral envelopes and intra-tegument protein-protein interactions would remain intact, and antibodies would not have access to tegument or capsid antigens. Incubation in H_2_O would lead to an osmotic rupture of the envelopes and increased epitope accessibility. Finally, an incubation in H_2_O followed by a high-salt KCl treatment weakened intra-tegument protein-protein interactions, thereby increasing tegument epitope accessibility, but possibly also leading to epitope denaturation and the extraction of tegument proteins.

After incubation in PBS, HSV-1 virions secreted from A549 cells expressing MxB(1–715) (Fig 6ai), MxB(26–715) (Fig 6aii), or MxA(1–662) (Fig 6aiii) were barely labelled by antibodies directed against Mx proteins and detected by protein A gold-particles (Fig 6d). On the other hand, MxB(1–715) (Fig 6bi), but neither MxB(26–715) (Fig 6bii) nor MxA(1–662) (Fig 6biii), were detected in the virions after osmotic shock by incubation in H_2_O (Fig 6d). However, the amount of accessible MxB(1–715) was reduced when the virions had been treated with 0.5 M KCl after the osmotic shock (Fig 6ci), either because MxB had been dissociated from the tegument or because the high salt treatment had denatured the MxB epitopes. However, after this treatment, more antibodies against Mx proteins were also bound to virions secreted from cells expressing MxB(26–715) (Fig 6cii) or MxA(1–662) (Fig 6ciii) suggesting that the KCl treatment might have exposed some unspecific cross-reactive epitopes (Fig 6d). While the labelling intensities of the virions treated with H_2_O followed by KCl were difficult to interpret, the immunoelectron microscopy data comparing the labelling intensities after PBS or H_2_0 treatment demonstrate that MxB(1–715), but neither MxB(26–715) nor MxA(1–662), was packaged into the tegument of HSV-1 virions (Fig 6d).

### The NTD of MxB, but not that of MxA, binds to alphaherpesvirus capsids

**Fig 7:**
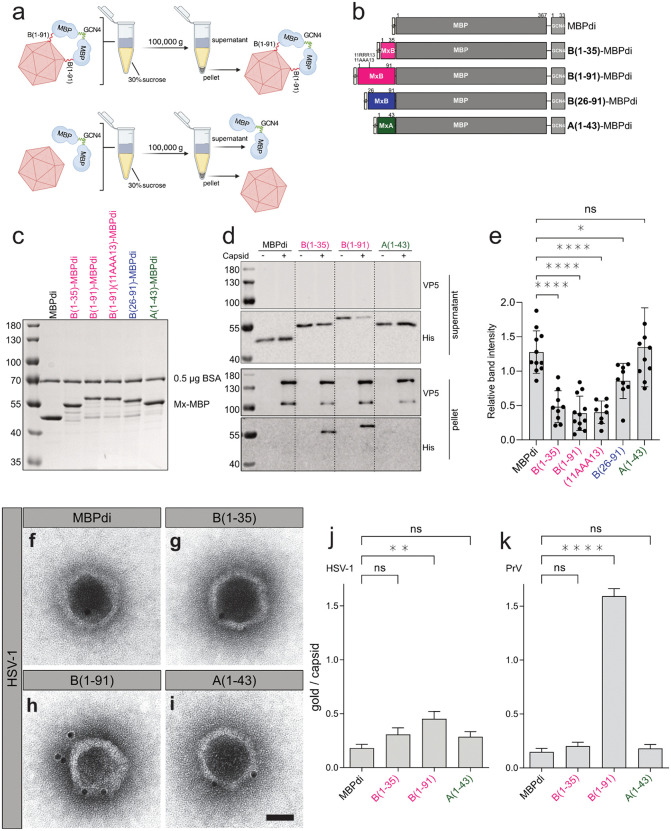
MxB-NTD, but not the MxA-NTD, binds to HSV-1 capsids. **(a)** Workflow of the capsid-host protein binding assay. HSV-1 or PrV capsids were incubated in the presence or absence of purified MBPdi proteins and sedimented at 100,000 × **g.** The capsids-host protein complexes and the respective supernatants were resuspended in sample buffer and analyzed by immunoblot (c, d, **e)**, or the capsids-host protein complexes were analyzed by immunoelectron microscopy (S2f-k Fig). Created with BioRender.com and licensed under CC BY 4.0 (https://BioRender.com/kfb4a8t). **(b)** Domain structure of MBPdi proteins with an N-terminal 6xHis-tag, empty, with in red MxB(1-91), in blue MxB-(26-91), or in green MxA(1-43), in dark grey the maltose-binding-protein (MBP, grey), and in light grey the GCN4 dimerization domain. **(c)** Lanes of a Coomassie-stained SDS gel showing the purified Mx fusion proteins of about 0.5 µg mixed with 0.5 µg of BSA. The markers on the left indicate the molecular weights. **(d)** Representative immunoblots from at least eight independent experiments of the supernatants (upper panels) or the pellets (lower panels) of MBPdi, MxB(1-35)-MBPdi, MxB(1-91)-MBPdi, or MxA(1-43)-MBPdi proteins incubated without (-) or with (+) HSV-1 capsids and probed with antibodies directed against the major capsid protein VP5 or the His-tag. **(e)** The mean intensities of the MBPdi proteins detected in the supernatants of the tegumented HSV-1 capsid-binding assays with anti-His antibodies were quantified using ImageJ. The relative band intensity was calculated by dividing the signals in the presence of capsids by the signals in the absence of capsids. Mean + /- SD from eight to 12 independent experiments. One-way ANOVA test with Dunnett multiple comparison: *, p < 0.05; **, p < 0.01; ***, p < 0.001; ****, p < 0.0001; ns, not significant. (**f, g, h, i**) Immunoelectron microscopy of de-tegumented HSV-1 capsids. De-tegumented HSV-1 capsids were adsorbed to EM grids and incubated with the MBPdi proteins for 60 min at room temperature, washed, and processed for immunoelectron microscopy using His-tag specific antibodies and 10 nm protein A-gold particles, and contrasted with uranyl acetate. Scale bars, 50 nm. **(j and k)** The mean number of protein A-gold particles within a 20 nm distance of 150 randomly documented HSV-1 (j) or PrV (k) capsids per condition, collected across 3 technical replicates, was determined. The error bars represent the SEM. Kruskal-Wallis test with Dunn’s multiple comparison: *, p < 0.05; **, p < 0.01; ***, p < 0.001; ****, p < 0.0001; ns, not significant.

HSV-1 capsids recruit MxB(1–715) and MxB(26–715) but not MxA(1–662) from cytosolic extracts [42]. Biochemical experiments have shown that HIV-1 capsid-like structures co-sediment with recombinant fusion protein dimers of the MxB NTD with the maltose-binding protein (MBP) and the dimerization domain (di) of the yeast transcription factor GCN4 [38]. We therefore test whether the NTD MxB(1–91) fused to MBPdi could also bind to HSV-1 or PrV capsids (Fig 7a). We cloned MBPdi constructs with an NTD of MxB(1–35), MxB(1–91), MxB(1–91)(11AAA13), MxB(26–91), or MxA(1–43) and a N-terminal His-tag (Fig 7b), expressed them in *E. coli*, and purified them on Ni-NTA affinity beads (Fig 7c). Moreover, we prepared tegumented HSV-1 capsids by lysing extracellular particles with the detergent TX-100 and weakening intra-tegument interactions with a treatment of 0.5 M KCl. Such capsid preparations contain capsid and inner tegument proteins, but proportionally much less outer tegument and envelope proteins than the starting material [39–42,60].

The MBPdi proteins were left in PBS or incubated with capsids, the samples were layered onto sucrose cushions, and the capsids were sedimented by ultracentrifugation (c.f. Fig 7a). The fractions above the sucrose cushions and the pellet fractions were analyzed by immunoblot using anti-His antibodies to detect the chimeric MBPdi proteins (Fig 7d). The capsids, as indicated by blotting for the major capsid protein VP5, were completely sedimented and not detected in the supernatants (Fig 7d). MBPdi protein alone and MxA(1–43)MBPdi remained in the supernatants and were not transferred to the pellet fractions, even when incubated with capsids (Fig 7d). In contrast, both, MxB(1–35)MBPdi and MxB(1–91)MBPdi co-sedimented with the capsids to the pellet fractions and were significantly reduced in the corresponding supernatant fractions (Fig 7d).

Unfortunately, we could not consistently and quantitatively resuspend the pellet fractions from ultracentrifugation tubes, either within or across experiments, without occasional contamination from the respective supernatants. Therefore, we quantified MBPdi signals from the supernatant fractions, as reported before [38]. MxB(1–35)MBPdi, MxB(1–91)MBPdi, MxB(1–91)(11AAA13)MBPdi, and to some extend MxB(26–91)MBPdi but not MxA(1–43)MBPdi were significantly depleted from the supernatant fractions by the addition of capsids when compared to the MBPdi control (Fig 7e). MxB(11AAA13) does not inhibits HIV-1 but still restricts HSV-1 infection [24], consistent with MxB(1–91)(11AAA13)MBPdi still binding to capsids (Fig 7e). Moreover, MxB(26–91)MBPdi bound less well than MxB(1–91)MBPdi to the capsids (Fig 7e), consistent with MxB(26–715) being less able than the full-length MxB(1–715) to restrict HSV-1 infection (c.f. Fig 1d).

Furthermore, we used immunoelectron microscopy to evaluate the association of MBPdi proteins with capsids. We prepared tegumented capsids from extracellular particles and treated them with TX-1000/KCl and trypsin to generate de-tegumented HSV-1 and PrV capsids, as reported previously for HSV-1 [42]. The capsids were adsorbed onto EM grids and incubated with MBPdi (Figs 7f, S3a, S3e), MxB(1–35)MBPdi (Figs 7g, S3b, S3f), MxB(1–91)MBPdi (Figs 7h, S3c, S3g), or MxA(1–45)MBPdi (Figs 7i, S3d, S3h). All grids were washed and incubated with rabbit anti-His antibodies, followed by colloidal gold coated with protein A. MxB(1–91)MBPdi bound to the HSV-1 capsids (Fig 7j), and even more so to the PrV capsids (Fig 7k), while MxB(1–35)MBPdi or MxA(1–45)MBPdi did not bind significantly better than MBPdi alone. Together, these experiments indicate that the NTD MxB(1–91), or even the shortened MxB(1–35) but not MxA(1–43), might suffice to recruit Mx proteins to the capsids of the alphaherpesviruses HSV-1 and PrV.

## Discussion

MxB(1–715) restricts the infection of alphaherpesviruses at low MOI, binds to their capsids, and induces capsid disassembly and premature release of genomes from capsids in biochemical assays and in cells. In contrast, the truncated MxB(26–715) isoform that lacks the N-terminal extension of residues 1–25 is less potent in restricting HSV-1 infection and inducing capsid disassembly [16,18,24,42]. Here, we focused on the role of the N-terminal domain (NTD; residues 1–91) of MxB in restricting infection of HSV-1 and PrV and binding to HSV-1 and PrV capsids.

We report that in addition to the human alphaherpesviruses HSV-1, HSV-2, and VZV, MxB(1–715) also restricted infection of the porcine alphaherpesvirus PrV at low MOI (Figs 1–3). Interestingly, the truncated isoform MxB(26–715) restricted PrV as effectively as the full-length MxB(1–715), whereas MxB(26–715) was less effective in restricting HSV-1. The molecular mechanisms underlying this differential susceptibility of PrV versus HSV-1 remain unclear, but this indicates that the NLS in the MxB(1–25) N-terminal extension contributes more to the restriction of HSV-1 than of PrV. This might be due to challenging a swine herpesvirus prepared in rabbit cells with human MxB proteins in human cells versus a human virus prepared in human cells and challenged with human MxB proteins in human cells.

Because MxB exerts its restriction at low MOI in multiple-step growth curves [16,24], we determined its subcellular localization and assessed whether MxB expression might alter viral particle assembly. Although we rarely detected progeny HSV-1 capsids co-localizing with MxB(1–715) molecular condensates (Fig 3), MxB(1–715) was packaged into the tegument of progeny HSV-1 and PrV particles. These data suggest that progeny capsids interact at low levels, transiently, or only with soluble MxB not sequestered into condensates. All of these may contribute to the challenge of detecting MxB on progeny capsids with the available antibodies by immunofluorescence microscopy.

Using quantitative mass spectrometry (Fig 4), our estimates of virion composition corroborate previous MS-based characterizations of particles assembled in BHK, HeLa, or retinal pigment cells [53,56,58]. We detected all major structural proteins and the packaging of several host proteins as reported before [56,58]. However, although the Mx proteins were expressed at similar levels, only MxB(1–715), but not MxA(1–662), was enriched in extracellular particles. Further immunoblot analyses indicate that the majority of particle-associated MxB(1–715) was protected by viral envelopes and co-sedimented with capsids isolated from extracellular HSV-1 or PrV particles (Fig 5). Thus, MxB fractionated like an inner tegument or capsid protein during TX-100 lysis at a high salt concentration [39,41,42,60].

To determine whether MxB might have self-assembled into filaments [11] and therefore sedimented into the capsid fraction, we analyzed secreted HSV-1 virions directly by immunoelectron microscopy (Fig 6). Anti-Mx antibodies revealed little background labeling when the samples had been maintained in PBS. But after a treatment with H_2_O to induce an osmotic rupture of the viral envelopes, there was a significant labeling on the virions released from MxB(1–715) but not from MxB(26–715) or MxA(1–662) cells, consistent with the notion that MxB had bound to tegument or capsid proteins during capsid envelopment into the virions. Unfortunately, in contrast to virion fractionation in solution [39–42,60], we could not recapitulate the high-salt tegument dissociation on the virions adsorbed onto the formvar film of the electron microscopy grids, as the treatment with 0.5 M KCl led to an increase in unspecific labeling of the three different preparations. Nonetheless, labeling was also higher in virions derived from MxB(1–715) A549 cells than in those derived from MxA (1–662) cells.

Tegumented and de-tegumented HSV-1 capsids recruit MxB(1–715), but to a lesser extent MxB(26–715) from cytosolic extracts [42], and the NTD residue M83 modulates the activity of human MxB against herpesviruses [17]. To evaluate whether the MxB(1–91) NTD could bind directly to capsids, we purified chimeric proteins consisting of the Mx NTDs fused to the maltose-binding protein and a dimerization domain (Fig 7). In co-sedimentation assays, MxB(1–91), MxB(1–91;11AAA13), and MxB(1–35), but not MxA(1–43), bound to tegumented HSV-1 capsids. MxB recognizes different molecular patterns on viral capsids, but in contrast to binding to HIV capsids [36,38], the MxB(11RRR113) motive did not contribute to HSV-1 capsid binding, consistent with MxB(1–715;11AAA13) restricting HSV-1 infection as efficiently as MxB(1–715) [24]. Furthermore, immunoelectron microscopy showed that MxB(1–91) but not MxB(1–35) bound to de-tegumented HSV-1 and PrV capsids. These data suggest that de-tegumented capsids lacked binding sites for MxB(1–35) but retained those for MxB(1–91).

In our protocol, outer HSV-1 tegument proteins, such as pUL11, ICP4, VP11/12, VP13/14, VP16, and VP22, are reduced on tegumented capsids, whereas inner tegument proteins, such as pUS3, pUL14, pUL16, pUL21, pUL36, pUL37, and ICP0, remain capsid-associated. In contrast, the so-called de-tegumented capsids used for immunoelectron microscopy (Figs 7, S3) contain very little tegument [40,42]. Thus, MxB might bind to tegument and/or capsid surface proteins, and possibly other capsid-associated host proteins late in infection to be packaged into virions during cytoplasmic capsid envelopment. As a result, MxB might end up in the virions bound to inner tegument and/or the capsid surface and/or by hitchhiking or piggybacking on capsid-associated host proteins.

Herpesviral capsids assemble in the nucleus and traverse the nuclear envelope to acquire further tegument proteins in the cytosol before final cytoplasmic envelopment (reviewed in Döhner et al. 2024 [31]). It will be interesting to investigate whether MxB induces capsid disassembly only during cell entry and nuclear targeting [18,42] or also late in infection. A sufficient number of capsids might egress from the nuclei for cytoplasmic capsid envelopment despite an ongoing MxB-induced capsid disassembly. Furthermore, herpesviruses might encode antagonistic tegument or non-structural proteins that shield the progeny capsids from the MxB activity. Accordingly, we showed that tegumented capsids are less susceptible to MxB-induced disassembly than de-tegumented capsids in cell-free assays [42].

Future studies should assess whether endogenous MxB, whose expression has been induced by interferon, is also packaged into the tegument of alphaherpesviruses and determine the specific infectivity of such MxB-loaded extracellular particles. In principle, the packaged MxB protein could exert antiviral or proviral functions. If MxB remained capsid-associated during microtubule transport and nuclear targeting (reviewed in [31,61]), it might support the docking of incoming capsids to nuclear pores, as MxB can bind directly to specific nucleoporins [14,18,19]. Alternatively, capsid-associated MxB might help repress viral replication by nucleating the assembly of MxB oligomers onto incoming capsids during their cytoplasmic transport. In this context, it is worth noting that MxB oligomerization is important for impairing nuclear targeting of incoming HSV-1 capsids [16,18,24,42].

Our study demonstrates that MxB employs similar mechanisms to inhibit herpesviruses as to restrict lentiviruses. We show that the MxB NTD is essential for binding to herpesviral capsids and for restricting herpesvirus replication. The cell lines and cell-free assays developed herein constitute robust platforms for defining the structural requirements of the MxB NTD and further elucidating the molecular basis of MxB’s antiviral activity. Future investigations will utilize recombinant MxB fusion proteins and protein domains as affinity baits to identify MxB interaction sites on the capsid surface and to investigate the impact of naturally occurring MxB single-nucleotide polymorphisms (SNPs) on potential herpesviral escape from MxB-mediated restriction.

## Materials and methods

### Cells

Hamster kidney BHK-21 (American Type Cell Collection CCL-10), rabbit kidney RK-13 cells (cell culture collection CCLV-RIE 109; Friedrich Löffler Institute, Insel Riems, Germany), African green monkey kidney Vero (CCL-81), and human lung epithelial A549 (CCL-185) were cultivated in Dulbecco’s modified Eagle’s medium (DMEM) supplemented with 10% fetal calf serum (FCS) at 37°C and 5% CO_2_. Human lung epithelial A549 cells expressing constitutively MxB(1–715), MxB(26–715), or MxA(1–662) under the control of the human cytomegalovirus immediate early promoter (Fig 1a) or mock-transduced were cultured in DMEM supplemented with 10% FCS and 2 µg/mL puromycin at 37°C and 5% CO_2_ as reported before [24,42].

### Biologicals

We used human IFNa2 (NBP2–34971, Novus Biologicals, Bio-Techne GmbH, Wiesbaden, Germany), and mouse monoclonal antibodies directed against a conserved epitope in the Mx GTPase domain (pan-Mx M143 [62], the His-tag (clone HIS-1 Sigma) HSV1-gD (DL6; sc-21719, Santa Cruz Biotechnology), HSV1-ICP8 (ab20194, Abcam), or HSV1-VP16 (clone G2 LP1) [63]. Moreover, we used rabbit polyclonal sera directed against MxA(506–573) (NBP1–83120, Novusbio), MxB(9–84) (NBP1–81018, Novusbio), or β-actin (Abcam), and rabbit monoclonal antibodies directed against MxA (ab207414, Abcam). Further rabbit sera were specific for PrV-pUL19 (VP5, [64], PrV-gB [65], PrV-pUL37 [66], PrV-pUL31 [67], HSV-1 capsids (VP5, SY4563 [68], HSV1-pUL11 [69], HSV1-pUL21 (from John Wills), HSV1-pUL25, HSV1-pUL37 [70], or HSV1-VP22 (pUL49) [71]. We used the secondary, fluorescently labeled goat anti-rabbit IRDye 800CW IgG (H + L), anti-rabbit IRDye 680RD, and anti-mouse IgG (H + L) IRDye 800CW (LI-COR BioSciences) antibodies, as well as goat anti-rabbit AF488 (Thermo Fisher Scientific, A32731) and goat anti-mouse AF 647 (Thermo Fisher Scientific, A32728).

### Viruses

We used the BAC-derived strain HSV1(17^+^)Lox (HSV-1 for short) [49], HSV1(17+)Lox-CheVP26 [49], and the PrV isolate Kaplan (PrV for short) [72]. To prepare virus inocula, extracellular particles were pelleted from the media of HSV-1-infected BHK-21 cells or PrV-infected RK-13 cells, resuspended in PBS, aliquoted, and stored at -70°C in single-use aliquots until further use [73]. The inocula were plaque-titrated by serial dilution on Vero cells for HSV-1 or on rabbit kidney RK-13 cells for PrV.

### Viral growth curves

A549 cells expressing MxB(1–715), MxB(26–715), or MxA(1–662) or mock-transduced A549 control cells were inoculated with 0.001 MOI of HSV-1 or with 0.0001 MOI of PrV. The cell culture supernatants were harvested at the indicated time points and plaque-titrated on Vero cells for HSV-1 or on rabbit kidney RK-13 cells for PrV.

### Immunoblot

Infected cells or viral particle fractions were solubilized in SDS sample buffer at 95°C for 5 min, and the proteins were separated by 10% or 20% SDS PAGE and blotted onto PVDF membranes (Merck). The membranes were incubated with blocking buffer with 0.1% [v/v] Tween-20, 5% [w/v] milk powder in PBS alone for 1 h, with the primary antibodies diluted in blocking buffer for 1 h at RT or at 4 °C overnight, and with washing buffer with 0.1% [v/v] Tween-20 in PBS for 3 x 10 min. After incubation with fluorescently labeled secondary antibodies diluted in blocking buffer for 1 h at RT, the membranes were washed, and the fluorescent signals on the membranes were detected using a fluorescent imager (LI-COR ODYSSEY Fc Imaging).

### Immunofluorescence microscopy

Cells infected with HSV1-CheVP26 were fixed with 3% [w/v] PFA in PBS for 20 min, the remaining fixative was quenched with 50 mM NH_4_Cl/PBS for 10 min, and the cells were permeabilized with 0.1% Triton X-100/PBS for 5 min. The HSV1-Fc receptor [74] and other unspecific protein binding sites were blocked with 0.5% [w/v] BSA and 10% [v/v] human HSV1-negative serum in PBS [75,76]. The cells were subsequently incubated with primary and secondary antibodies diluted in blocking solution. DNA was stained by 10 µg/mL 4′,6-diamidino-2-phenylindole (DAPI) in PBS with 10% [v/v] DMSO, 0.1% [v/v] NP-40, 5% [w/v] bovine serum albumin, 10 mM Tris-HCl (pH 7.4), 150 mM NaCl, 2 mM CaCl_2_, and 2 mM MgCl_2_. The cells were embedded in antifading mounting medium (Immunoselect, #SCR-038447, Dianova) and documented by confocal fluorescence microscopy using plan-apochromat 63x/1.4 oil immersion objectives, and 405-, 488-, 561-, and 639-nm laser lines (LSM980 Airyscan with operating software ZEN Blue 3.2; Carl Zeiss, Jena, Germany). Contrast and brightness were adjusted identically across each set of images. Figures were assembled using Adobe Illustrator CS4 (version 14.0).

### RT-qPCR

A549 cells were cultured for 24 h in 6-well plates and treated for 24 h with 1,000 U/mL of human IFN-α2 (NBP2–34971, Novus Biologicals, Bio-Techne GMBH, Wiesbaden, Germany), or infected with HSV-1 at an MOI of 0.01 for 24 or 48 h. The cells were washed with PBS, and total RNA was extracted using 350 μL/well RA1 buffer supplemented with 3.5 μL β-mercaptoethanol, isolated using an RNA kit (NucleoSpin; Macherey-Nagel, Düren, Germany; REF 740955.50), and eluted in 60 μL/well H_2_O. cDNAs were synthesized from 1,000 ng RNA in 12 μL H_2_O using a reverse transcription kit (QuantiTect, Qiagen, Germany; Cat. No. 205311) with the reverse transcription step extended to 30 minutes at 42°C and diluted in Milli-Q water to 100 μL. For the PCRs, 5 μL of diluted cDNAs were combined with 5.5 μL of SYBR™ Green PCR Master Mix (Applied Biosystems, Cat. No. 4309155) and 0.5 μL of an 8 μM primer mix. The PCR reactions were performed in 384-well plates and analyzed using the QuantStudio 5 System (Applied Biosystems). To detect HSV-1 ICP0 transcripts, we used the sense primer 5’-CGACCCTCCAGCCGCATACGA-3’ and reverse primer 5’-TTCGGTCTCCGCCTGAGAGTC-3.’ To detect host mRNAs, we used customized primer pairs from Qiagen for γ-actin (Hs_ACTG1_1, QT00996415), IFN-β (Hs_IFNB1_1, QT00203763), IL-6 (Hs_IL6_1, QT00083720), IRF-7 (Hs_IRF7_1, QT00210595), ISG1 (Hs_ISG15_1, QT00072814), MX1 (Hs_Mx1_1, QT00090895), and MX2 (Hs_MX2_1_SG, QT00000581). The mRNA amounts of HSV1-ICP0, IFN-β, IL-6, IRF7, ISG15, MX1, and MX2 were normalized to γ-actin by the 2^-ΔCT^ method [77], and presented as mean values ± SD of biological triplicates with each dot representing the mean of technical duplicates. Statistical significance was determined using one-way ANOVA with Dunnett’s correction (*p < 0.05, **p < 0.01, ***p < 0.001, ****p < 0.0001).

### Preparation of extracellular HSV-1 and PrV particles from cells expressing Mx proteins

A549-MxB(1–715), A549-MxB(26–715), or A-549-MxA(1–662) cells seeded at 1.5 x 10^7^ per 150 mm dish and cultured for 1 d were inoculated with HSV-1 at an MOI of 0.001 or with PrV at an MOI of 0.0001 in DMEM without FCS for 2 h at RT. The cells were cultured in DMEM with 3% FCS, 20 mM HEPES, pH 7.5, and 0.05% [w/v] NaHCO_3_ at 5% CO_2_ and 37°C for 48–72 h until strong cytopathic effects had developed. The supernatants were harvested and pre-cleared by centrifugation at 1,800 x g for 20 min, and the extracellular particles were sedimented at 100,000 x g for 90 min at 10°C (SW32 rotor), resuspended in PBS, aliquoted, snap-frozen in liquid N_2_, and stored at -80°C. Moreover, the infected cells were harvested, resuspended in PBS, aliquoted, snap-frozen, and stored at -80°C for later protein analyses.

### Mass spectrometric proteome analyses of extracellular HSV-1 particles and infected A549 cells

For all mass spectrometry analyses, the samples were digested in solution.

Extracellular particles harvested from the medium of A549-MxB(1-–715) or A549-MxA(1-–662) cells infected with HSV-1, as described above, were thawed, resuspended in PBS to about 1 x 10^9^ PFU, loaded onto 9 mL glycerol-tartrate gradients and centrifuged at 111,000 x g for 60 min at 10°C without brake (SW41 rotor, Beckman) as described before [54]. The upper bands containing light particles (L particles) lacking capsids and genomes, and the lower bands of heavy particles (H particles) containing intact virions, were aspirated separately. The particles were washed twice by sedimentation at 100.000 x g for 45 min at 10°C (TLA-55 rotor, Beckman) and resuspension in PBS. The L and H particles were resuspended in 100 µL PBS and stored in aliquots at -70°C. The particles were lysed by adding urea to a concentration of 8 M and triethylammonium bicarbonate (TEAB) to 50 mM and shaking for 30 min at 4°C. The samples were supplemented to 5 mM Tris(2-carboxyethyl)phosphine hydrochloride (TCEP) and 40 mM chloroacetamide (CAA) and incubated in the dark at RT for 1 h.

HSV-1-infected A549 cells were thawed and lysed in 7 M urea, 1% Triton X-100, 5 mM TCEP, 30 mM CAA in 50 mM TEAB. After adding 700 U/mL benzonase (Merck), the samples were incubated on ice for 30 min and sonicated at 4°C for 45 min (30 sec on, 30 sec off; Bioruptor Pico, Diagenode). The proteins were extracted using methanol-chloroform precipitation and dried [78].

Endoproteinase Lys-C was added at an enzyme-to-substrate ratio of 1:75 for 3 h at RT to digest the proteins. The samples were diluted with 50 mM TEAB to a concentration of 2 M urea, further digested with trypsin overnight at an enzyme-to-substrate ratio of 1:100, desalted using Stage tips [79], and resuspended in 1% acetonitrile (ACN) and 0.05% trifluoracetic acid. Reverse-phase separation was performed on a vanquish neo system using an in-house-packed C18 column (Poroshell 120 EC-C18, 2.7 μm particle size, Agilent Technologies) at 250 nL/min with increasing ACN concentration.

All fractions were analyzed on a mass spectrometer (Orbitrap Exploris 480, Thermo Scientific; Instrument Control Software version 4.2) in data-dependent acquisition (DDA) mode using the parent-ion mass scan (MS1) with a resolution of 120,000, scan range at 375–1200 m/z, custom AGC target at 300% and automatic maximum injection time mode. The peptides were fragmented with higher-energy collisional dissociation (HCD) at 30% normalized collision energy. For the fragment-ions scan (MS2), a resolution of 15,000, an isolation window of 1.6, and a standard AGC target were used. A 2 second time between master (MS1) scans was set.

The mass spectrometry data were analyzed with the software MaxQuant (version v1.6.2.6) using trypsin/P protease specificity, carbamidomethylation on cysteine as a static, oxidation on methionine, as well as N-terminal acetylation as variable modifications, and enabling the options LFQ (label-free-quantitation), iBAQ (intensity-Based Absolute Quantification), and match between runs [80–82]. The false-discovery rate (FDR) was set to 1% at the PSM (peptide spectrum matches), protein, and modification site level. We based their identification only on unique peptides for independent quantification of the rather homologous MxA and MxB proteins.

### Fractionation of extracellular HSV-1 and PrV particles

Tegumented V_0.5_ capsids were prepared as reported before [39–42]. Extracellular particles harvested from the medium of infected A549-MxB(1–715), A549-MxB(26–715), or A549-MxA(1–662) cells were resuspended in 0.6 mL PBS to about 3.3 x 10^8^ PFU/mL for HSV-1 or 6.7 x 10^7^ PFU/mL PFU for PrV, and sedimented through a 0.4 mL cushion of 30% [w/v] sucrose in PBS*.* HSV-1 or PrV particles containing about 3 x 10^9^ PFU in 0.35 mL were incubated with 1 U/µL trypsin (Sigma, Darmstadt) at 37°C for 40 min to digest any host or viral proteins attached to the particle surfaces. To stop the proteolysis, protease inhibitors (cOmplete cocktail, Roche Diagnostics) were added to a final volume of 0.4 mL. The viral particles were lysed by adding 400 µL of 2 x lysis buffer with 1 M KCl, 2% [v/v] Triton X-100, 20 mM DTT, 20 mM HEPES, 30 mM Tris, pH 7.4, and 75 U / mL of benzonase (Merck, Darmstadt) and incubated on ice for 30 min. The 0.8 mL samples were added to 0.5 mL 30% sucrose cushions in PBS and pelleted at 4°C and 100,000 x g for 45 min (TLA-55 rotor, Beckman). The pooled pellets of the V_0.5_ capsids were washed with PBS, resuspended in 0.25 mL capsid-binding buffer with 5% [w/v] sucrose, 20 mM HEPES-KOH, pH 7.3, 80 mM K^+^ acetate, 10 mM DTT, 1 mM EGTA, 2 mM Mg^2+^ acetate. Equivalent samples of each step of the fractionation protocol were resuspended in 60 µL SDS sample buffer and analyzed by immunoblot.

### Preparation of Mx-NTD fusion proteins

Maltose-binding protein (MBP) expression constructs were cloned into pSKB-LNB-based on pET28 backbone [9]. MBP-GCN4 cDNA was PCR amplified from pET-MxB(1–35)-MBPdi [38] and cloned upstream of the His-tag and the PreScission cleavage site of pSKB-LNB using EcoRI and XhoI. In addition, a NheI site was inserted in front of the MBP cDNA. In this basic MBP-GNC4 construct the cDNAs coding for the N-terminal Mx domains were inserted in frame to the MBP ORF using the NdeI and NheI restriction sites, resulting in plasmids, pSKB-LNB-His-MBP-GCN4, pSKB-LNB-His-B(1–35)-MBP-GCN4, pSKB-LNB-His-B(1–91)-MBP-GCN4, pSKB-LNB-His-B(26–91)-MBP-GCN4, and pSKB-LNB-His-A(1–43)-MBP-GCN4.

His-Mx-MBP-GCN4 proteins were expressed as N-terminal His-tag fusion proteins from the pSKB-LNB-His-Mx-MBP-GCN4 vector in *E. coli* Rosetta cells (DE3, Novagen 70954, Genotype: F^-^ ompT hsdSB(rB^-^ mB^-^) gal dcm (DE3) pRARE (Cam^R^)). The bacteria were cultured in lysogeny broth medium at 20°C for 6 h. At an optical density of 0.2 at 600 nm, protein production was induced by adding 0.02 mM IPTG for 12 h at 20°C. The bacteria were harvested by sedimentation, washed in PBS and lysed by ultrasonication in 50 mM Tris, pH 8.0, 500 mM NaCl, 20 mM imidazole, 7 mM β-mercaptoethanol, and EDTA-free protease inhibitors (cOmplete cocktail, Roche Diagnostics). The cleared lysates were incubated with Ni-NTA agarose (Qiagen, Hilden), washed twice, and eluted with 20 mM Tris (8.0), 100 mM NaCl, 250 mM imidazole, 5% glycerol, and 7 mM β-mercaptoethanol as described [83]. His-tagged protein-containing fractions were pooled, dialyzed against 20 mM Tris, pH 7.5, 100 mM KCl, 20% glycerol, 0.2 mM EDTA, 1 mM DTT for 16 h at 4°C, and concentrated to about 1 µg/µL protein using centrifugal filter units (Amicon Ultra, 30 kDa, Millipore, Darmstadt). Aliquots of the purified fusion proteins were stored at -70°C: MBPdi, MxB(1–35)-MBPdi, MxB(1–91)-MBPdi, MxB(26–91)-MBPdi, and MxA(1–43)-MBPdi. The protein concentrations of the lysates were determined by the Bradford assay (Bio-Rad, USA), and their compositions were analyzed with SDS-PAGE followed by Coomassie blue R-250 staining using BSA as a standard.

### Preparation of de-tegumented HSV-1 and PrV capsids

BHK-21 cells seeded at 1.5 x 10^7^ per 150 mm dish and cultured for 1 d were inoculated with 5 mL DMEM containing HSV-1 at an MOI of 0.001 PFU/cell and RK13 cells with PrV at an MOI of 0.0001 PFU/cell on a rocking platform. After 2 h at RT, 12 mL of DMEM with 4% FCS were added, and the dishes were transferred to a 5% CO_2_ incubator set at 37°C. When cytopathic effects had developed, the supernatants from the infected cells were collected and clarified by low-speed centrifugation at 1,800 x g for 20 min. The extracellular particles were sedimented at 100,000 x g for 90 min at 8°C (SW32 rotor, Beckman), and resuspended in 200 µL PBS, loaded onto a 500 µL cushion of 30% glycerol in PBS, and centrifuged at 100.000 x g for 45 min at 8°C (TLA-55 rotor, Beckman). The resulting pellets were resuspended in 300 µl PBS, aliquoted, and stored at -70°C.

To prepare de-tegumented, digested D capsids, the extracellular viral particles were thawed and resuspended in 2 x lysis buffer at a final concentration of 1% [v/v] Tx-100 and 100 mM KCl, and tegument proteins were dissociated by limited trypsin digestion at 10 U/ml for 35 min at 37°C as reported before [42]. The capsids were sedimented through a 500 µL cushion of 30% sucrose in PBS at 100,000 x g at 4° for 45 min. They were resuspended in capsid-binding buffer (5% [w/v] sucrose, 20 mM HEPES-KOH, pH 7.3, 80 mM K^+^ acetate, 10 mM DTT, 1 mM EGTA, 2 mM Mg^2+^ acetate) to a concentration of 1 x 10^7^ capsid equivalents/mL. As reported before, the capsids were used for co-sedimentation assays or directly absorbed onto grids used for electron microscopy analyses [42].

### Mx protein co-sedimentation with viral capsids

Before incubation with the viral capsids, the purified Mx-MBPdi fusion proteins were diluted with capsid-binding buffer (CBB; 20 mM HEPES, pH 7.4, 80 mM K-acetate, 2 mM Mg-acetate, 1 mM EGTA, 2 mM DTT, 5% glycerol) to a final concentration of about 0.1 µg/µL. The diluted proteins were then incubated for 30 min at 37°C and centrifuged in a TLA-55 rotor at 100,000 x g for 45 min at 4°C. Then, 180 µl of the pre-centrifuged Mx-MBPdi supernatants were mixed with 50 µl of the HSV-1 capsids or just with 50 µl 1xCBB as a control, and incubated for 30 min at 37°C. Then, the mixtures were loaded onto a 500 µl 30% sucrose cushion in CBB and centrifuged again at 100,000 x g for 45 min at 4°C. The pellets were resuspended in 40 µl of 1 x SDS sample buffer. Aliquots of the UC supernatants and pellets were analyzed by immunoblot using a histidine-specific antibody and the anti-VP5 antibody, detecting the capsids. The Western blot signals of the Mx-MBPdi protein bands in the supernatant fractions were quantified using ImageJ, and the relative band intensities were calculated by comparing the band intensities of the anti-His signals after centrifugation in the presence and the absence of viral capsids.

### Immunoelectron microscopy

Extracellular particles secreted from HSV-1 infected A549-MxB(1–715), A549-MxB(26–715), or A-549-MxA(1–662) cells were thawed and resuspended in PBS. They were treated with 50 U/mL benzonase at 37°C for 30 min, with 5,000 U/mL trypsin at 37°C for 30 min, and with 5 mg/mL soybean trypsin inhibitor (SBTI; Fluka, Switzerland) for 10 min on ice. The particles were adsorbed onto enhanced hydrophilicity 400-mesh formvar-carbon-coated copper electron microscopy grids (Stork Veco, The Netherlands) for 20 min at RT. The grids were transferred on 20 µL droplets of PBS (with 137 mM NaCl) for 2 x 30 min (control in Fig 6), of H_2_O for 30 min followed by PBS for 30 min (osmotic shock in Fig 6), or of H_2_O for 30 min followed by 500 mM KCl for 30 min (osmotic shock + high salt in Fig 6). The grids were washed with PBS and labeled with the pan-Mx-specific monoclonal M143 antibody [62] at RT for 1 h followed by protein-A gold (10 nm, Cell Microscopy Centre, Utrecht, Netherlands) for 30 min at RT.

For the *in-vitro* binding studies (Figs 7F-7I and S3), the grids were placed on 20 µL droplets of de-tegumented D capsids at 1 x 10^7^ capsid equivalents/mL for 20 min as reported before [42]. The grids with the adsorbed capsids were then placed on droplets with different MBPdi proteins at 0.05 µg/µL in CBB at 37°C for 60 min. The grids were washed with PBS, labelled with the anti-His tag-specific mouse monoclonal antibodies for 1 h and secondary rabbit anti-mouse antibodies (Cappel™, MP Biomedicals, USA) for 30 min followed by protein-A gold for 20 min.

After the immunolabeling, the grids were washed with PBS and H2O, contrasted with 2% uranyl acetate at pH 4.4, dried, and analyzed by transmission electron microscopy (Morgani, Eindhoven, Netherlands) as reported previously [40,42,84]. The number of gold particles per capsid was counted for 100 (Fig 6) or 150 (Figs 7f-7k and S3) capsids per condition. Labelling of capsids incubated with control IgG instead of anti-His antibody was considered background and subtracted. Samples lacking primary antibodies served as controls and did not yield any labeling with Protein A gold.

### Data, analyses, and presentation

For analysis, we loaded the proteingroups.txt output into the software Perseus (version v2.0.7.0) and removed protein contaminants, reverse database hits, and proteins identified only by modification sites [81,82]. iBAQ (intensity-based absolute quantification) values for all three H-particle replicates were summed up and log-transformed. For statistical analyses, we performed unpaired t-tests comparing H particles (3 replicates) to cell levels (4 replicates) using Perseus. Relative protein quantifications were discarded when not quantified in all cellular and particle replicates. Plots were generated using in-house generated R/Rstudio scripts. The other numeric data were analyzed with GraphPad Prism 8.4.2, the schematic drawings were created with BioRender.com, and the figures were assembled with Affinity Publisher 1.9.2.1035.

## Supporting information

S1 Fig Subcellular localization of Mx proteins during HSV infection and interaction with enveloped progeny capsids. A549 cells were infected with HSV1(17^+^)Lox-CheVP26 at an MOI of 20, fixed at 9 hpi, and labeled for MxB or MxA (green), glycoprotein gD (purple), and DNA (blue). Representative images of cells expressing empty control, MxA, MxB(1–715), or MxB(26–715). The arrows indicate single progeny capsids in the cytoplasm. Panels **(e)** and **(f)** show two representative examples of cells expressing MxB(1‑715) illustrating the asynchronous infection kinetics among different cells. Scale bar: 1 µm.
(TIF)

S2 Fig Expression profiles during HSV-1 low MOI infection. A549 cells were mock-treated (control), treated with human IFN-α a2 at 1000 U/mL for 24 h, or infected with HSV-1 at an MOI of 0.01 for 24 or 48 h. (**a - e, g, h**) The expression of HSV1-ICP0 (infected cell protein 0), and host IFN-β (interferon beta), IRF7 (interferon-response factor), Mx1, Mx2, ISG-15 (interferon-stimulated gene 15), and IL-6 (interleukin 6) were quantified relative to actin by the 2^-ΔCT^ method. Means ± SD of triplicates, with each dot representing the mean of technical duplicates. One-way ANOVA test with Dunnett multiple comparison: *, p < 0.05; **, p < 0.01; ***, p < 0.001; ****, p < 0.0001; ns, not significant. (**f**) Cell lysates were separated by SDS-PAGE, transferred to PVDF membranes, and probed with antibodies against the Mx GTPase domain (panMx M143), MxA, MxB, or VP5 (pUL19). Actin was used as a loading control. Molecular weight markers are indicated on the left.
(TIF)

S3 Fig The NTD of MxB binds to HSV-1 capsids. Immuno-EM analysis of Mx-MBPdi bound to tegument-free HSV-1 (a-d) and PrV (e-h) capsids. Pre-adsorbed de-tegumented capsids were incubated with purified Mx-MBPdi proteins for 60 min at room temperature as indicated. Then the grids were washed and further processed for immunogold labeling using the His-tag specific antibody. Protein A gold particles were scored as one bound-Mx protein if localized within 20 nm of the capsid and as multiple copies if the gold particles were at a distance of ≤ 20 nm. Scale bars represent 50 nm.
(TIF)
