# Supplementary figures and images for: The antiviral GTPase MxB is packaged into virions and binds via its N-terminal domain to alphaherpesvirus capsids
Source: PLoS Pathog. 2026 Jul 16;22(7):e1014370. doi: 10.1371/journal.ppat.1014370 (PMC13374926; doi:10.1371/journal.ppat.1014370)

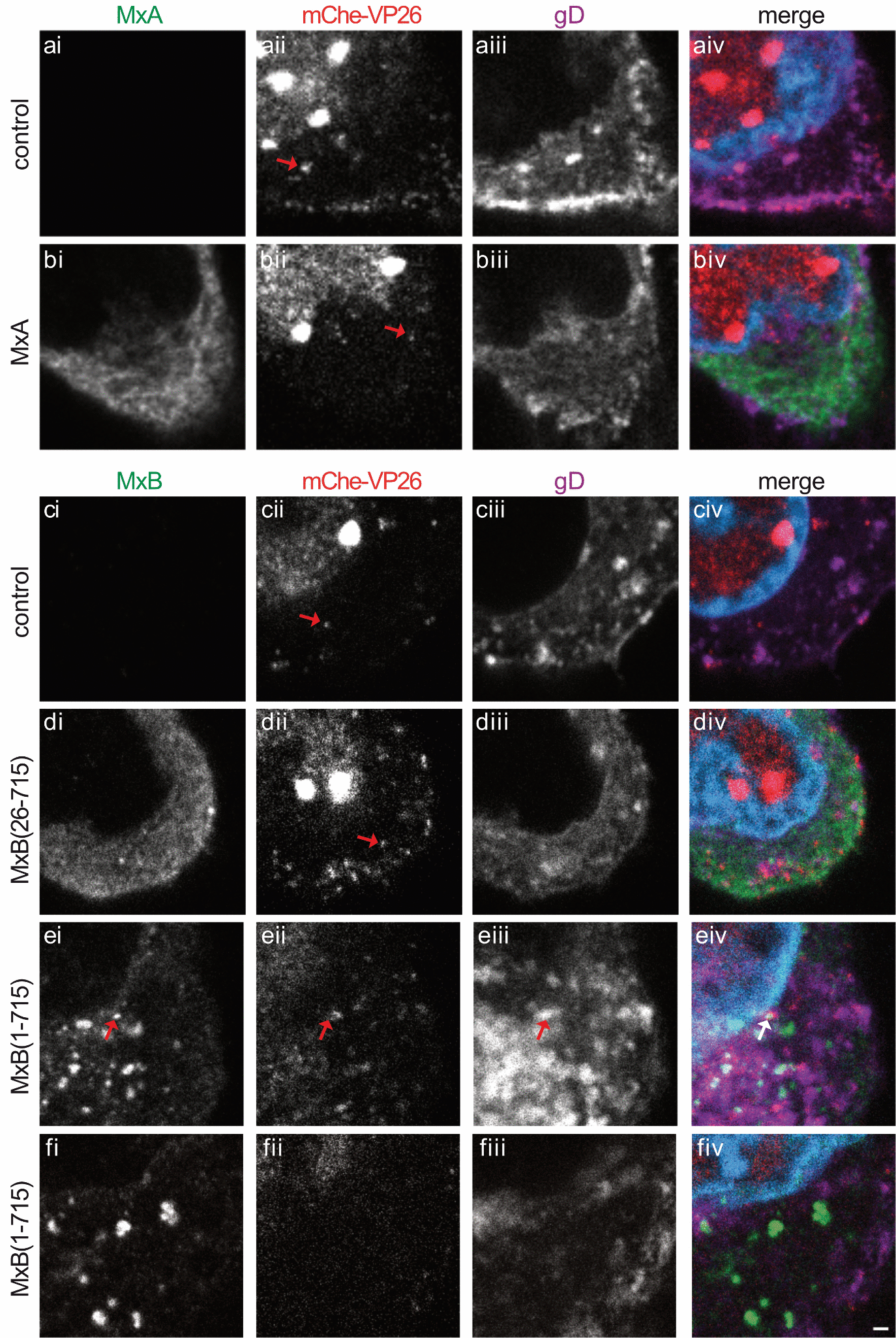

Supplement: S1 Fig — A549 cells were infected with HSV1(17+)Lox-CheVP26 at an MOI of 20, fixed at 9 hpi, and labeled for MxB or MxA (green), glycoprotein gD (purple), and DNA (blue). Representative images of cells expressing empty control, MxA, MxB(1–715), or MxB(26–715). The arrows indicate single progeny capsids in the cytoplasm. Panels (e) and (f) show two representative examples of cells expressing MxB(1‑715) illustrating the asynchronous infection kinetics among different cells. Scale bar: 1 µm. (TIF) [file ppat.1014370.s001.tif]
